# “The communication and support from the health professional is incredibly important”: A qualitative study exploring the processes and practices that support parental decision‐making about postmortem examination

**DOI:** 10.1002/pd.5575

**Published:** 2019-11-04

**Authors:** Celine Lewis, Megan Riddington, Melissa Hill, Charlotte Bevan, Jane Fisher, Lucy Lyas, Ann Chalmers, Owen J. Arthurs, John C. Hutchinson, Lyn S. Chitty, Neil Sebire

**Affiliations:** ^1^ North East Thames Regional Genetics Service Great Ormond Street Hospital for Children NHS Foundation Trust London UK; ^2^ Genetics and Genomic Medicine The UCL Great Ormond Street Institute of Child Health London UK; ^3^ Department of Psychological Services Great Ormond Street Hospital for Children NHS Foundation Trust London UK; ^4^ Stillbirth and neonatal death charity (Sands) London UK; ^5^ Antenatal Results and Choices (ARC) London UK; ^6^ The Lullaby Trust London UK; ^7^ Child Bereavement UK Cheshire UK; ^8^ Department of Radiology Great Ormond Street Hospital for Children NHS Foundation Trust London UK; ^9^ Department of Histopathology Great Ormond Street Hospital for Children NHS Foundation Trust London UK; ^10^ Development Biology and Cancer Programme The UCL Great Ormond Street Institute of Child Health London UK

## Abstract

**Background:**

Consent rates for postmortem (PM) examination in the perinatal and paediatric setting have dropped significantly in the United Kingdom, the United States, and the Western Europe. We explored the factors that act as facilitators or barriers to consent and identified processes and practices that support parental decision‐making.

**Methods:**

A qualitative study conducted with bereaved parents, parent advocates, and health care professionals in the United Kingdom. Analysis was conducted on 439 free‐tect comments within a cross‐sectional survey, interviews with a subset of 20 survey respondents and 25 health professionals, and a focus group with five parent advocates.

**Results:**

Three broad parental decision‐making groups were identified: 1, “Not open to postmortem examination”; 2, “Consent regardless of concerns”; and 3, “Initially undecided.” Decisional drivers that were particularly important for this “undecided” group were “the initial approach,” “adjustment and deliberation,” “detailed discussion about the procedure,” and “formal consent.” The way in which these were managed by health care staff significantly impacted whether those parents' consented to PM, particularly for those who are ambivalent about the procedure.

**Conclusions:**

We propose a set of recommendations to improve the way PM counselling and consent is managed. Adopting such measures is likely to lead to improved family experience and more consistent and high‐quality discussion regarding PM.

## Abstract 2

What's already known about this topic?

- There has been a significant decline in uptake of paediatric post mortem, despite evidence that it provides clinically useful data in between 22% and 76% of cases.

What does this study add?

- This study identifies key actions by health care staff that are highly influential in whether or not parents' consent to the postmortem procedure. Recommendations for practice are also provided.

**What's already known about this topic?**

- There has been a significant decline in uptake of paediatric post mortem, despite evidence that it provides clinically useful data in between 22% and 76% of cases.

**What does this study add?**

- This study identifies key actions by health care staff that are highly influential in whether or not parents' consent to the postmortem procedure. Recommendations for practice are also provided.

## 1 BACKGROUND

In the United Kingdom, around one in 80 pregnancies results in either termination following diagnosis of a fetal anomaly, stillbirth, or neonatal death representing at least 8000 cases per annum, and there are over 500 unexplained infant and childhood deaths annually.^1^, ^2^, ^3^ In such cases (excluding those where the death is referred to HM Coroner, and parental consent for post mortem is not required), parents may be offered a postmortem examination (PM), also known as autopsy, to try to establish the cause of death and, where appropriate, estimate risk of problems reoccurring in future pregnancies.^4^ Despite the fact that such perinatal and paediatric PM has been shown to provide useful clinical data in between 22% and 76% of cases,^4^ there has been a significant decline in uptake globally in recent years.^5^, ^6^, ^7^, ^8^, ^9^ Data from the United Kingdom from 2017 show that only 45% of parents of stillborn babies and 26% of parents of neonates who died consented to PM examination.^10^ Parental dislike of the invasiveness of the procedure, poor communication between professionals and parents about the procedure, ambivalence about the value of the procedure from health professionals themselves, and religious objections have been identified as key barriers to uptake.^11^

The way in which PM is offered in the United Kingdom has changed in recent years. In 2013, Sands, the stillbirth and neonatal death charity, launched the Sands Post Mortem Consent Package, which was developed to provide information and guidance about PMs for health professionals as well as families.^12^ This included major changes to the hospital consent form, which was reorganised with parents' priorities in mind, made shorter in some cases than other existing forms at the time, and importantly marked a shift away from medicalised terminology to clearer, kinder, and more accessible wording. In 2016, the Human Tissue Authority (HTA) introduced codes of practice for PM examination.^13^ This included the consent process containing clear guidance on options for how tissue may be handled after the PM examination; training for health professionals responsible for seeking consent; and regular assessment of competency to ensure skills were maintained. Less invasive methods of PM have also been developed including the use of imaging techniques, in particular magnetic resonance imaging (MRI), which can also be used to guide further tissue‐sampling techniques.^14^, ^15^, ^16^

As part of a National Institute for Health Research Health Technology Assessment project (14/168/06), the authors conducted a mixed methods research programme to look at key stakeholders' views, experiences, and attitudes towards different methods of PM including less invasive approaches. Stakeholders included bereaved parents,^17^ health professionals,^18^ HM Coroners,^18^ and religious groups.^19^ During that research, we collected data on the processes and practices that support parents with decision‐making and the factors that act as facilitators or barriers to parental consent, which we present in this manuscript.

## 2 METHODS

### 2.1 Ethical approval

National Health Service Research Ethics Committee approval for this study was obtained in April 2016 (16/LO/0248 from London‐Bloomsbury Ethics Committee).

### 2.2 Study design and recruitment

This qualitative study conducted in the United Kingdom comprises data gathered from a cross‐sectional survey, interviews, and a focus group. For a more detailed account of the study design and recruitment, please refer to our published papers^17^, ^18^, ^19^ and Data S1.

#### 2.2.1 Cross‐sectional survey with bereaved parents

A survey (Data S2) exploring parental views towards different types of PM was developed specifically for this study. Following each set of questions, participants were invited to provide free‐text comments explaining their response. At the end of the survey, participants could choose to provide contact details if they wished to take part in a telephone interview. The survey was made available through the online survey website SurveyMonkey (Survey Monkey Inc, Palo Alto, California, USA) as well as in paper format. Anyone who had experienced the loss of a pregnancy (either through miscarriage and termination of pregnancy for a fetal abnormality) or had experience of a perinatal or infant death was eligible to complete the survey irrespective of whether they had been offered a PM or a PM had been requested by the coroner's office.

Bereaved parents were recruited both retrospectively and prospectively between June 2016 and December 2017. Retrospective recruitment was through the social media channels, eg, Facebook, of four support groups that support parents who have experienced termination for fetal anomaly, stillbirth, neonatal, infant, or child death. Potential participants were directed to the online survey. Prospective recruitment was conducted through seven hospitals across England. Women and their partners who were 18 years of age and over and had experienced loss of pregnancy (as described above) were eligible to participate in the study. Participants were recruited into the study by a member of the health care team following the autopsy examination discussion, irrespective of whether they consented or declined. Potential participants were briefly informed about this study, and if they were interested in taking part or finding out more, given a study pack containing the survey.

#### 2.2.2 Qualitative interviews and focus groups

Survey responders who indicated their willingness to take part in an interview were purposively sampled to ensure a range in terms of their loss, whether they consented to a PM procedure or not, and demographics. No time limit was set in terms of how many months prior to the interview the loss occurred. Health professionals across the United Kingdom from a range of clinical backgrounds whose roles include being involved in discussions with parents about PM examination or conducting or interpreting PM results were identified by the authors, purposively sampled, and invited via email to participate in the study. A focus group was conducted with five parent advocates from the four support groups involved in retrospective recruitment for the survey.

Interviews and focus groups explored parents' experience of being approached about PM including what support they received when making a decision (for those for whom a Coroner's PM was not required), health professionals' experience of discussing PM with parents, reasons that parents accept or decline PM, and their information and support needs. Interviews were conducted by MR or CL between April 2016 and July 2017. For all interviews and focus groups, written consent was sought to digitally record the discussions, transcribe them verbatim, and use anonymised quotes.

### 2.3 Data Analysis

Analysis of qualitative data (free‐text comments, interview, and focus group transcripts) was conducted following principles of thematic analysis, with themes derived from the data^20^ and supported by Nvivo version 10 (QSR International, Pty Ltd). The data from all sources were analysed as a single data set. The first transcripts were coded independently by M.R., C.L., or M.H. and a coding framework agreed. Coding and analysis was then conducted by M.R., C.L., or M.H. with a subset coded by at least two researchers to ensure inter‐rater reliability. Coding was compared, and any disagreements were discussed until consensus was reached. The research team met at regular intervals to review themes and to generate a thematic “map” of the analysis. Final themes were reviewed and agreed by M.R., C.L., and M.H. Data collection (for interviews) continued until saturation was reached. In the final stage of the project the findings were presented to patient representatives and health professionals at a dissemination meeting to check for accuracy and resonance with their experiences (member checking).

## 3 RESULTS

### 3.1 Participants

In total of 938 surveys were returned: 870 through retrospective recruitment (655 Sands, 108 ARC, 81 Lullaby Trust, and 26 Child Bereavement UK) and 68 through prospective recruitment (30% recruitment rate); 79 were excluded due to missing data leaving 859 for inclusion in the analysis (Table 1). Of those 859, 439 (51%) included free‐text comments for analysis. Thirty‐six survey responders were contacted to take part in an interview, and 20 consented and took part (56% response rate) (Table 1). For the health professional interviews, 40 health professionals were contacted, and 25 took part, from 11 different hospitals (63% recruitment rate) (Table 2).

**Table 1:** Bereaved parent demographics

	Survey Sample N = 859 % (n)	Interview Participants N = 20 % (n)
Age	Range, 18‐73; Mn, 35.9; Mdn, 35.0; SD, 8.1	Range, 25‐64; Mn, 39.6; Mdn, 37.0; SD, 9.5
Sex		
Female	94.9% (615)	90% (18)
Male	2.7% (23)	10% (2)
Country of birth		
United Kingdom	94.5% (774)	85% (17)
Other	5.5% (45)	15% (3)
Education		
No formal qualification	1.7% (14)	/
GCSE or equivalent	21.5% (177)	5% (1)
A level or equivalent	24.4% (201)	15% (3)
Degree or equivalent	32.8% (271)	45% (9)
Postgraduate qualification	19.5% (161)	30% (7)
Ethnicity^a^		
White or White British	95.0% (783)	85% (17)
Black or Black British	2.5% (21)	5% (1)
Asian or Asian British	1.3% (11)	5% (1)
Mixed	0.6% (5)	5% (1)
Other	0.5% (4)	/
Do you have a religious faith?		
Yes	48.2% (393)	55% (11)
No	51.8% (423)	45% (9)
If YES, which faith?^b^		
Christian	44.8% (358)	73% (8)
Muslim	0.8% (6)	/
Jewish	0.8% (6)	27% (3)
Sikh	0.5% (4)	/
Hindu	0.4% (3)	/
Jehovah's Witness	0.4% (3)	/
Buddhist	0.1% (1)	/
Experience of loss (tick all that apply)		
Miscarriage (loss up to 12 wk' gestation)	34.3% (295)	25% (5)
Late miscarriage/fetal loss (12‐24 wk' gestation)	18.7% (161)	15% (3)
Stillbirth	47.4% (407)	45% (9)
Termination for fetal anomaly	18.3% (157)	20% (4)
Neonatal/infant death (0‐12 mo)	22.0% (189)	35% (7)
Child death (1‐16 y)	2.3% (20)	5% (1)
None	0% (0)	0% (0)
Approached about autopsy		
Yes	83.2% (711)	75% (15)
No	7.4% (63)	5% (1)
Not sure	2.1% (18)	
Coroner requested an autopsy	7.4% (63)	20% (4)
Consented to autopsy		
Yes	67.1% (485)	73% (11)
No	32.9% (238)	27% (4)

**Table 2:** Health professional participants

Total Participants	25
Profession	
Bereavement midwife	6
Anatomical pathology technologist	4
Intensive care consultant	4
Obstetrics/fetal medicine consultant	4
Perinatal/paediatric pathologist	3
Intensive care unit family liaison nurse	2
Consultant neonatologist	1
Paediatric radiologist	1

### 3.2 Qualitative findings

We begin by reporting the context in which decisions about PM examination are made and outline three broad parental decision‐making groups that were identified. Finally, we explore the “steps towards decision making” taken by parents and highlight the decisional drivers that acted as either barriers or facilitators to consent for the group that were initially undecided about PM. Example quotes are indicated in the text, eg, Q1, and provided in Tables 3, 4, 5, 6.

**Table 3:** Landing in unexpected territory, example quotes

Theme	Example Quote
Landing in unexpected territory	Q1: “I remember trying to comprehend what the doctors were saying, but at that time your head is so overwhelmed by grief.” *Sands questionnaire participant #300 – declined PM (free‐text comment)*
	Q2: “You're dividing your brain between the intellectual response which is ‘yes, I should have this done because it will tell me why my baby died' and the emotional response which is ‘this is horrendous, I can't believe I have to go through this'. And I think therefore the communication and support from the health professional is incredibly important.” *Parent advocate ‐ focus group*
Group 1: Not open to postmortem examination	Q3: “I see the importance of an autopsy but my view is the child and parents have suffered enough through the loss. Nothing will ever bring the child back. Let sleeping babies sleep in peace.” *Sands questionnaire participant #602 – declined PM (free‐text comment)*
Group 2: Consenting regardless of concerns	Q4: “We wanted the best chance of determining cause of death which was a higher priority than maintaining the integrity of our child.” *Hospital questionnaire participant #L33 – consented to PM*
	Q5: “I wanted to know what the hell had I done, had anybody done. Was he born with something strange, did he catch something because it was a hot day I had all the windows open, what on earth went wrong?” *Lullaby Trust interview #39 – consented to PM*
Group 3: Initially undecided	Q6: “Now we are forever questioning if there was something wrong that could have been detected, something we can do to prevent it from happening again or something we did that we need to be aware of for future pregnancies. I now know autopsy is extremely important to have answers and feel closure.” *Sands questionnaire participant #496 – declined PM (free‐text comment)*

**Table 4:** The initial approach, example quotes

Theme	Example quote
Routinely approaching everyone	Q7: “The main reason uptake it not so good is because not all families are approached in the first place.” *Family liaison nurse 1*
	Q8: “If I knew that a family had strict Muslim views I probably wouldn't offer the PM because, you know, I've never had someone say yes and I think often it causes quite significant distress.” *Consultant neonatologist 1*
	Q9: “How it was said was just like in passing ‘do you want a cup of tea', ‘no', ‘OK then right', that's how I felt it was, you know.” *Sands interview #35 – declined PM*
Timing	Q10: “You go to sit with a woman, you talk to the woman, you gauge where she's at, you discuss what she wants to discuss and you put the post‐mortem in at a point that they're ready to receive it.” *Bereavement midwife 5*
	Q11: “After the loss of a baby in this way it was very distressing to be asked if you want an autopsy within a few hours of the birth.” *ARC questionnaire participant #87 – declined PM*
	Q12: “I didn't have an autopsy and I really regret that now … and I think the main thing I want to get across is I was asked a matter of hours after [name] passed away. And I really do think that was very, very too soon.” *Sands interview #35 – declined PM*
	Q13: “The time spent describing autopsy happened before giving birth. It was difficult to comprehend what was going to happen and have time to reflect so it was easier to say no.” *Sands questionnaire participant #581 – declined PM (free‐text comment)*
Having an established relationship with staff member making the approach	Q14: “The midwives spend more time with them, they build up a rapport with them and then it's easier for them to talk about post‐mortem … I think they are the best people to talk about it and that will increase the percentage of uptake.” *Fetal medicine consultant 1*
Dedicated facilities and staff	Q15: “I think because we were on the labour ward, they just wanted to get rid of us as quickly as possible, for our own benefit. [...] They didn't want us sitting on a ward – there's people giving birth around us – they didn't quite know what to do with me. That's the impression I got.” *Sands interview #44 – consented to PM*

**Table 5:** Detailed discussion about the procedure, example quotes

Theme	Example quote
Consent as a conversation	Q16: “I don't think that it's fair for the health professionals to shy away from those conversations, because of their fears, when actually, it can potentially have such a massive effect [...] because actually, the parents – we've been through the worst thing we can possibly go through.” *Child Bereavement UK interview #55 – consented to (limited) PM*
	Q17: “If somebody had sort of come in and said a bit brusquely ‘we need to do this, we need to do that' I would probably have said ‘you're not touching him' … But somebody literally sat on the bed and took a lot of time … we had an awful lot of time spent with us.” *Sands questionnaire participant #38 – consented to PM*
Specialist staff conveying confidence and empathy	Q18: “I was helped by a bereavement midwife who explained fully where my baby would go and what would happen way before I had to consent to a PM, but she put my mind at ease.” *Sands questionnaire participant 91 – consented to PM (free‐text comment)*
	Q19: “From a nursing point of view a lot of the bedside nurses wouldn't have the confidence to ask about post mortem because they don't know exactly what's involved and the parents may ask some questions that they don't have answers for.” *Family liaison nurse 1*
	Q20: “[..] the midwife will come to the door and say [to the doctor], ‘Oh, no, it's OK. This family don't want a postmortem.' And you can almost see a lot of doctors say, ‘Oh, thank God for that. I don't have to go and have that discussion'.” *Obstetrician 1*
Perceived added value of PM	Q21: “A lot of our parents … will say something like ‘the midwife said I shouldn't consent because it's not worth it and it's very unlikely to tell me anything', so you are very influenced by the professional who's consenting you.” *Patient advocate ‐ focus group*
	Q22: “We were advised that it was very doubtful they would find any reason other than prematurity as cause of death.” *Sands questionnaire participant #188 – declined PM (free‐text comment)*
	Q23: “I think some parents where the death was maybe a cord round the neck and they kind of want to accept that as the cause of death. We do say to them that it might not have been, but they say no, we've seen it and that's what it is.” *Bereavement midwife 3*
	Q24: “My son died at 41 weeks with no problems throughout the pregnancy, he was born with the cord round his neck and he had messed his waters, this was enough of a reason for me.” *Sands questionnaire participant #230 – declined PM (free‐text comment)*

**Table 6:** Formal consent, example quotes

Theme	Example quote
Consent forms should be sensitively worded	Q25: “That's a pretty horrible bit of paperwork in the consent process about bits of tissues and receiving slides and organs at home, which you have to do.” *Consultant ICU 1*
Too much information about the process can cause distress to parents	Q26: “The detailed description the consultant insisted on giving us despite requests not to do it nearly stopped us consenting and still causes distress. We knew what it meant but didn't need the detail.” *Sands questionnaire participant #340 – consented to PM*
	Q27: “With the Sands consent form the wording of it is just more straightforward, it's a little bit softer, it acknowledges the fact that it is somebody's baby.” *Bereavement midwife 1*

### 3.3 Landing in unexpected territory

The loss of a baby or child was a traumatic experience, which catapulted parents into unexpected territory. Parents reported experiencing high levels of emotional distress and “shock.” They felt vulnerable and in some cases noted that absorbing complex information at such a difficult time was challenging. For some, the physical pain and ongoing effects of labour medication were also present (Q1). Decision‐making about PM was found to be an added burden for parents at a time when they were experiencing a huge amount of grief.

When asked to make a decision about PM, parents tended to fall into one of three decisional groups;

Group 1 Those parents who were not open, under any circumstances, to a PM (“Not open to postmortem examination”);
Group 2 Those parents for whom the need for answers overrode any concerns about the procedure (“Consenting regardless of concerns”); and
Group 3 Those parents who were ambivalent towards PM and for whom careful management and counselling by the health professional team was key in supporting them towards the *right choice for them* (“Initially undecided”) (Q2).

#### 3.3.1 Group 1: Not open to postmortem examination

For some parents, the “brutal” and “horrific” invasiveness of the PM procedure and concerns around “leaving the child looking like a rag‐doll” were too much to bear and created a barrier to consent. There was a strong parental drive to protect the baby from “harm” and to let the child “rest in peace.” PM was seen as furthering physical and psychological harm to the parent and baby/child without any prospect of “bringing the child back” (Q3). For some parents, their reason for declining PM was driven by their religious beliefs.

#### 3.3.2 Group 2: Consenting regardless of concerns

For other parents, there was an “absolute need to know” what led to the loss of their baby/child in order to have answers and to prevent it from happening again. This overrode dislike of the invasiveness of the procedure (Q4). In a minority of cases, “mismanagement” and medical negligence were queried, but more commonly, parents wanted a PM to ensure they were not to blame for their baby's death (Q5). For some parents who had terminated a pregnancy because of a fetal anomaly, they wanted reassurance that the diagnosis was “accurate” and validation that they had made the “right” choice. Some parents spoke of “owing it” to their child to find out what happened. For some parents, altruistic reasons such as contributing to research and preventing this “awfulness” from happening to other parents were important.

#### 3.3.3 Group 3: Initially undecided

The third group of parents were those who were undecided about PM in the initial period after the loss. For this group, getting it right in terms of supportive systems and structures was found to be key in facilitating personalised decision‐making. When communication was poor and support lacking, parents were more likely to decline PM, a decision that some parents later regretted (Q6).

### 3.4 Steps towards decision‐making

**Figure 1:**
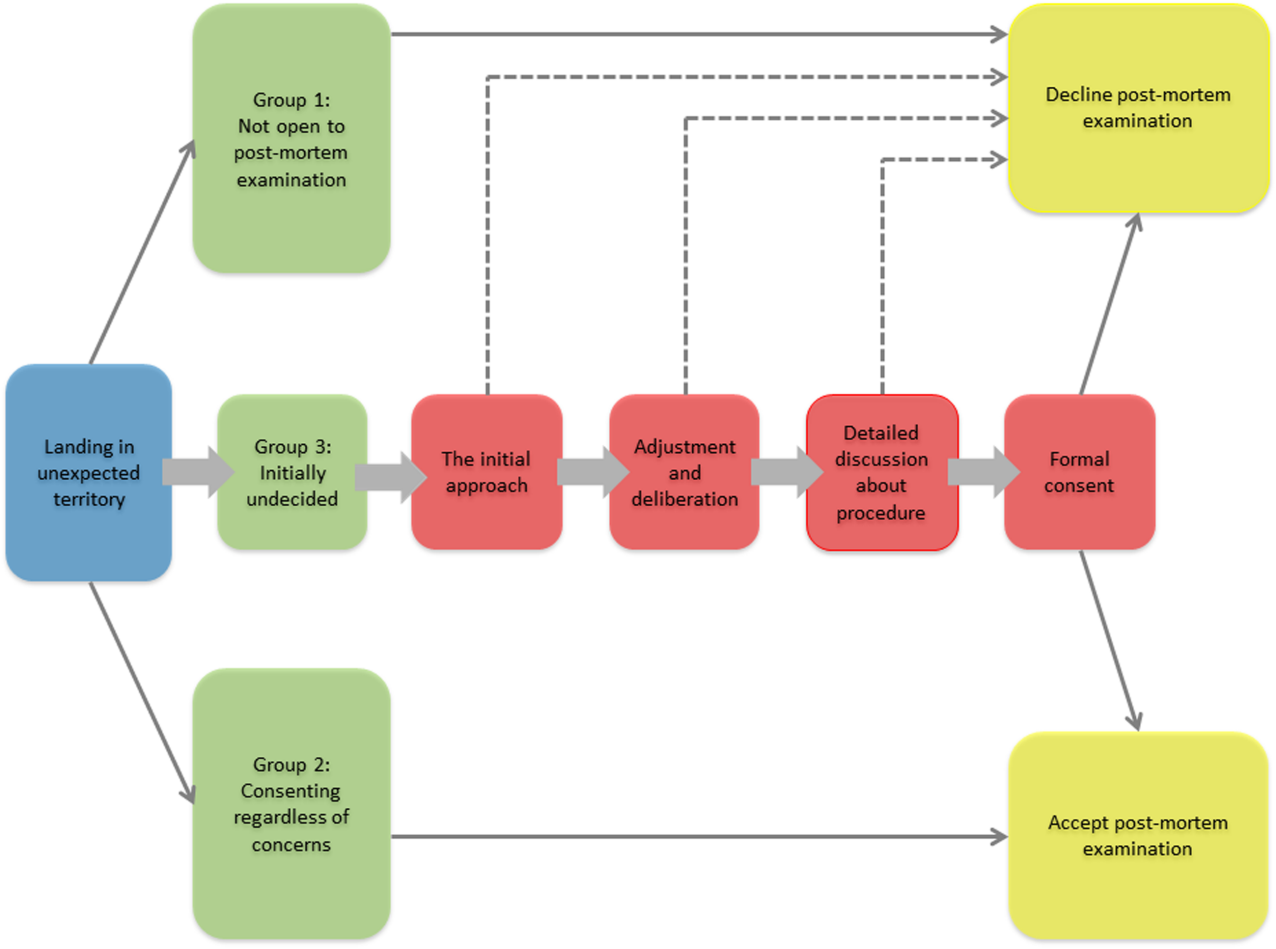
Decisional drivers for postmortem [Colour figure can be viewed at http://wileyonlinelibrary.com]

Through our analysis, we identified a series of four decisional drivers that were particularly important for this “undecided” group (Figure 1). The way in which these were managed by health care staff significantly impacted whether or not those parents' consented to PM. While we acknowledge that the consent process may not always occur in this way, we believe this is a helpful way to think about decision‐making for PM.

#### 3.4.1 The initial approach

“Planting the seed” for PM commonly fell to frontline health professionals such as midwives and obstetricians, who were already involved in the care of the parents or child. The way that the initial approach was managed significantly impacted whether parents were open to considering the procedure.

#### 3.4.2 Routinely approaching everyone

Routinely asking every family about autopsy was identified as “good practice” by health professionals and something staff “ought to do” (Q7). Nevertheless, health professional stereotypes and preconceptions existed that meant not all parents were offered PM. Perception of parents' ethnicity or religion was found to influence the initial approach about PM. Professionals, in some cases, made assumptions about what parents would or would not want based on previous experiences, as well as the desire to be respectful and not increase parents' distress (Q8).

Emotional connections that had developed with families, particularly in the care of neonates or children in paediatric intensive care, were also found to prevent some health care professionals from raising the subject of PM because of a desire to protect those families from any further distress. There was also evidence that PM was sometimes briefly mentioned as a “tick‐box” exercise rather than a core component of ongoing care, which was more likely to result in parents declining (Q9).

#### 3.4.3 Timing

Sensitively timed approaches about PM were recognised as extremely important (Q10). Some parents who declined PM spoke about being asked too soon after their loss or at an inappropriate time, including at a time where both parents were not present (Q11). Raising the subject too soon when parents felt “utterly overwhelmed, emotional and physically weak” could lead to a “knee‐jerk” reaction to decline, which some parents later regretted (Q12). While participants noted that there “is never a good time” to be asked, a number of parents who had experienced stillbirth commented that they were asked about PM during labour or very soon after birth, which “seemed really insensitive” (Q13).

#### 3.4.4 Having an established relationship with staff member making the approach

Health professionals identified that it was good practice to have the initial approach made by someone with a pre‐existing relationship with a family where a level of trust had been established (Q14). Hospital chaplains of different faiths whom parents could speak to, to help guide decision‐making, were also seen as valuable in supporting parents in their decision.

#### 3.4.5 Dedicated facilities and staff

Dedicated facilities “solely for baby loss,” which were separate from the labour and postnatal wards where new mothers and babies were situated, provided an environment that was conducive to a sensitive initial approach being made. Not having access to such facilities was disturbing for both staff and parents (Q15).

## 4 ADJUSTMENT AND DELIBERATION

### 4.1 Allowing parents time to consider their decision

Allowing parents' time to consider their decision was seen as important by health professionals and parents. In some cases, health professionals conducted follow‐up phone calls or house visits in order to give parents time to adjust to the idea and ask further questions about PM if they needed to:

Sometimes I'll mention it and say “look I know you said you're not ready to talk about it, but can we give you a ring in a week? … So they kind of know it's coming. (Bereavement midwife 5)

### 4.2 Provision of written information

The provision of written information that parents could digest at their own pace and at a time when they were ready to think about it was also seen as important. Accessible material for parents to read was valued by those who received it; however, not all did. Leaflets/booklets from support charities providing information that was “fully explained” but presented in a way that was “a little bit softer” and a counterpoint to the “wordy,” “medical speak” of some consent documents.

## 5 DETAILED DISCUSSION ABOUT THE PROCEDURE

Many parents were unfamiliar with what a PM entailed, with a number commenting that their understanding of the procedure was shaped by “watching TV series.” Thus, it was incumbent on health professionals to overcome any misconceptions parents had about the procedure. It was recognised that PM procedure was “hard to describe in a way that people can feel pleasant about,” yet parents acknowledged the importance of health professionals having those conversations, with some feeling let down when it was not discussed in detail at a time when they needed “guidance” from people they could “trust” (Q16).

### 5.1 Consent as a “conversation”

Participants positioned consent at its best as “a conversation” where “time” was spent “talking through” the process and allowing adequate space for questions and consideration before signing the consent forms (Q17). Health professionals recognised their “personal opinion [was] irrelevant” and that parents should not be “influenced by the professional who's consenting [them].” Nonetheless, parents looked towards health professionals to guide them in their choice and desired communication that was competent, “open,” and “honest.” Being sensitive to the needs of individual families in terms of the amount and level of detail parents required to make an informed decision, heeding both verbal and nonverbal communication, was perceived as key.

### 5.2 Specialist staff conveying confidence and empathy

Some parents had clear memories of specially trained staff (eg, specialist nurses, bereavement midwives, and anatomical pathology technicians) offering “sensitive,” “professional,” “compassionate but never patronising” care. Being cared for by a knowledgeable, confident, and empathic health professional reassured parents their child was in safe hands during the PM procedure (Q18).

Treating PM as an integral part of the patient journey, where the patient would be cared for as if he or she were alive, was extremely valuable for parents. Reassurance around babies being treated with “dignity” and “respect,” being explicit that “great care [is taken with] with babies” and addressing concerns around “disfigurement” so that customs and rituals around death (eg, washing/dressing and final goodbyes) could still take place was found to be helpful. Additionally, naming the pathologist, positioning them as “part of” the “team,” and explaining how they work, was found to be beneficial. Some health care professionals reflected that colleagues were “squeamish,” felt “uncomfortable,” or experienced “fear” about discussing a process they felt ill‐informed about and considered to be “medical” and that this was a barrier to discussing the procedure (Q19).

Formal training for health professionals discussing PM with parents was found to be lacking, with a senior health professional commenting: “I don't think I've ever done a role play of a post mortem consent conversation.” Parents also acknowledged that “health professionals must get better at explaining the issues.” An initial decline by parents was found to be a barrier to any further in‐depth discussion with a more senior colleague (Q20). One health professional commented that while parents may initially decline when first asked, it can be worth revisiting that decision at a later point to minimise the risk that parents make a decision they later regret.

### 5.3 Perceived added value of PM

Health professionals noted that it was important that both parents and health professionals understood that PM was the most valuable tool available to establish cause of death and inform recurrence risk. Where health professionals were ambivalent about its value or discussed the procedure in a way that was dismissive of getting a diagnosis, this could lead to parents declining (Q21‐22). In cases where parents were ambivalent about the value of a PM, they were more likely to settle for a “plausible explanation” for the death and decline the procedure (Q23‐24).

## 6 FORMAL CONSENT

### 6.1 Consent forms should be sensitively worded

Both parents and health professionals described some consent forms as “a pretty horrible bit of paperwork,” “so long,” “unwieldy,” “graphic,” and “medicalised” (Q25).

### 6.2 Too much information about the process can cause distress to parents

There was an inherent tension between ensuring parents had enough information to give informed consent for the procedure and at the same time acknowledging that some parents do not want to go into a great amount of detail. Some parents who wanted a PM commented that they did not necessarily want the level of detail the form included (Q26). Health professionals also recalled families having declined PM at this stage, saying “I can't cope with that amount of detail, I'll leave it.” Some of the more recent consent forms such as the Post Mortem Consent Package developed by the support group Sands and endorsed by the Human Tissues Authority were acknowledged to be an improvement on older versions as they were worded more thoughtfully (Q27).

## 7 DISCUSSION

**Figure 2:**
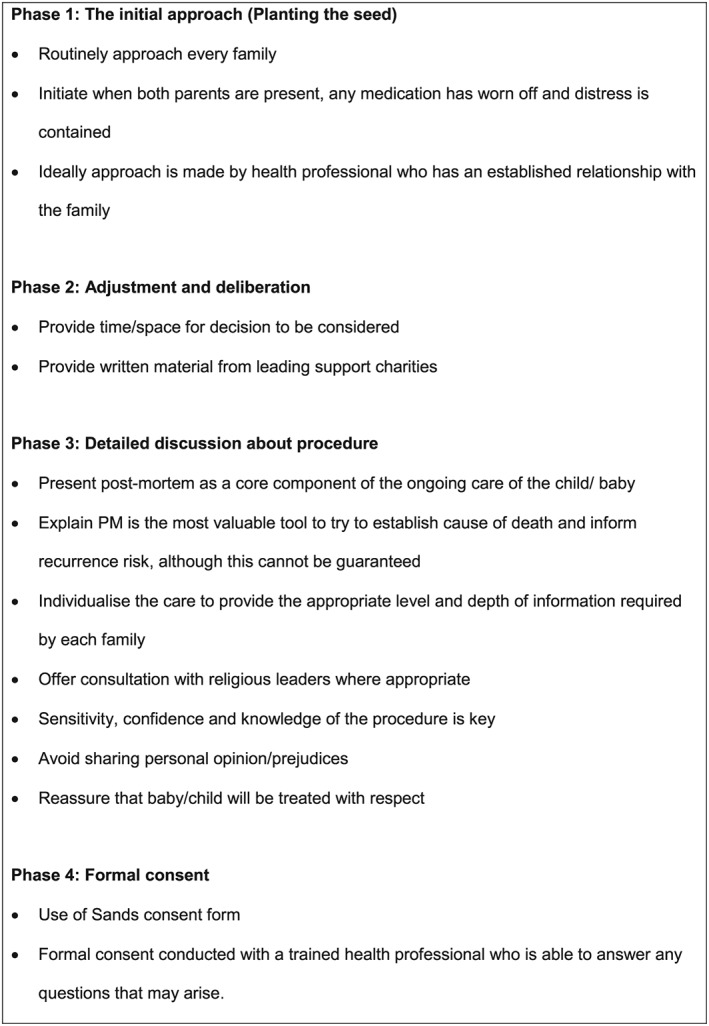
Recommendations for practice

The findings from this research are drawn from one of the largest studies on the experience of parental consent for PM that has been conducted in the United Kingdom.^17^ Our findings suggest that while some bereaved parents are very clear about whether PM examination is “right” for them at the time of approach, there are a significant number who do not have a strong opinion and for whom the actions of health care staff caring for them at that time are highly influential. During each phase of the counselling and consent process, we identified numerous health professional behaviours that either facilitated or hindered consent. This finding concurs with a key finding from the INSIGHT study focused on parental experience after stillbirth, where the authors identified that parents were highly influenced by discussions with staff.^21^ A similar finding was reported in a qualitative study conducted in Ireland where health professionals were found to play a key role, particularly if they can address parental concerns regarding the invasiveness of the procedure.^22^ The findings from this research are valuable in supporting recommendations for practice (Figure 2), which can be used to improve the way PM counselling and consent is managed.

Timing of the initial approach was found to be a key factor in whether parents consented to PM. Similar findings have been reported elsewhere.^22^, ^23^, ^24^ A number of parents in our study commented that the topic had been raised when they were in labour or had just given birth, something which is explicitly advised against in the Sands guide for consent takers.^12^ Inappropriate timing of the initial discussion about PM may also result in parents declining for purely emotional reasons, a finding which Meaney et al refer to as “rather than thinking with their head, they are thinking with their heart.”^22^ Time for parents to deliberate was also identified as an important component of good patient care in our study. A number of health behaviour theories, such as the theory of planned behaviour, consider deliberation of the available information to be a key component of the decision‐making process.^25^ Moreover, deliberation is seen as integral to informed decision‐making.^26^ As such, it is not surprising that when parents felt sufficiently informed and had adequate time to consider the pros and cons of the procedure, they were less likely to make decisions that they later regretted.

The three decisional groups that we identified in this study are supported by previous work on PM decision‐making conducted by Snowdon et al.^27^ In that study, the authors found that some parents were clear that they did not want a PM, others felt they needed the information from the PM, and then there was those parents who were initially discomforted by the idea but who then made the decision to go ahead.^27^ Our data suggest that many parents who decline PM do so early on in the discussion process, particularly if the timing of the approach is poor. When parents are supported in their decision‐making by empathetic staff and have time to deliberate, they are more likely to consent.

A commonly cited barrier to consenting to PM that was identified in both this study and others relates to parental dislike of the invasiveness of the procedure.^22^, ^28^, ^29^ In this study, we also found some parents were concerned about how their child would look after the procedure. The majority of participants in this study were not given the option of a less invasive PM since this is a newly emerging technique. Further research to examine real‐world uptake rates will be required as and when less invasive options become more routinely available to parents, to assess whether less invasive options do address one of the current key barriers to consenting.

Finally, since this study was conducted, there has been a great deal of work to improve communication and support surrounding PM consent. Sands Lothians have developed the animation “Parent to Parent Post Mortem Authorisation” (https://vimeo.com/272820256), which has been created with parents to dispel some of the myths surrounding PM procedure and to ensure parents have clear and accurate information to enable them to make an informed decision about PM. In addition, NHS Education for Scotland has developed training videos specifically to support staff breaking bad news as well as to have discussions around PM examination. These are available at http://www.sad.scot.nhs.uk/. The new National Bereavement Pathway currently being piloted in England and Scotland also includes professional guidance around consenting for PM at any stage of loss and is available at http://www.nbcpathway.org.uk.

### 7.1 Study limitations

The majority of surveys were collected retrospectively, and we do not know how long ago participants were asked about PM. We acknowledge that processes and procedures for consenting parents may have changed during this time. Participants were disproportionately UK‐born, highly educated, and White and a higher proportion consented to PM than the national uptake rates. Thus, the findings from this research may not be representative of the general UK population. Finally, participants who completed the survey were self‐selecting, and as a result, there may be responder bias. Nevertheless, these data include responses from a large group of bereaved parents including several clinical scenarios and as such represent an important and unique dataset in the field.

## 8 CONCLUSION

Parents' decision to undertake a PM for their child is significantly influenced by the quality of the interaction with the health care professional at the time, particularly for those who are ambivalent about the procedure at the time of the initial approach. Through this research, we have identified areas of good and poor practice and made recommendations as to how the interaction between parents and health care professionals can be improved. Adopting such measures is likely to lead to improved family experience and more consistent and high‐quality discussion regarding options for examination after perinatal and child death.

## FUNDING SOURCES

This work was supported by a National Institute for Health Research (NIHR) Health Technology Assessment, grant number 14/168/02. OJA is supported by a NIHR Clinical Scientist Fellowship award (NIPR‐CS‐012‐002), and NJS and LC are NIHR Senior Investigators. NJS and JCH are supported by Great Ormond Street Hospital Children's Charity. LSC and NJS are partially funded by the NIHR Biomedical Research Centre at Great Ormond Street Hospital. This article presents independent research funded by the National Institute for Health Research (NIHR). The views expressed are those of the author(s) and not necessarily those of the NHS, the NIHR or the Department of Health.

## AUTHORS' CONTRIBUTIONS

C.L. designed the survey instrument and interview questions, implemented the study, conducted qualitative interviews, analysed the data, and drafted and revised the paper. M.R. conducted qualitative interviews, analysed the data, and revised the draft paper. M.H. analysed the data and revised the draft paper. C.B., J.F., L.L., A.C., O.A., and J.C.H. interpreted the data and revised the draft paper. L.S.C. designed the study, interpreted the data, and revised the draft paper. N.J.S. designed the study, interpreted the data, and revised the draft paper.

## CONFLICTS OF INTEREST

None declared.

## Supporting information

Data S1. Supporting Information

Data S2. Supporting Information

## Data Availability

Full transcripts are not available due to issues of confidentiality; however, select passages will be available from the corresponding author on request.
