# Supplementary material for: “The communication and support from the health professional is incredibly important”: A qualitative study exploring the processes and practices that support parental decision‐making about postmortem examination
Source: Prenat Diagn. 2019 Nov 4;39(13):1242–53. doi: 10.1002/pd.5575 (PMC6973141; doi:10.1002/pd.5575)
Supplement: Supplementary file 1 — Data S1. Supporting Information [file PD-39-1242-s001.docx]

APPENDIX S2
Questionnaire
Principal Researcher: Professor Neil Sebire
Version 4. 9.6.16
IRAS 198952

Code number						
Recruiting site						

Personalising examination after death to improve experience for bereaved parents

Questionnaire
Confidential

Thank you for agreeing to take part in this study. Following the loss of a child or baby, many parents want an answer to the question ‘why did this happen?’, and ‘will it happen again?’ This is common whether the loss occurred during pregnancy, after birth or following termination of pregnancy for an anomaly. In order to try and answer these questions, doctors may think it would be useful to perform an investigation known as an autopsy examination (or post mortem). The aim of this questionnaire is to discuss with you a number of different methods of autopsy currently available and identify which method is preferable to you and why.

This questionnaire will take around 10-15 minutes to complete. Most of the questions just need you to tick a box. Please try to answer all the questions, but if you are unable to answer all of the questions, any information you can provide will be incredibly valuable in helping us gain a better understanding of these issues from a parent’s perspective.

Description of complete (or standard) autopsy examination

- *A complete autopsy examination involves a procedure like an operation in which all parts of the body can be examined in detail, including the internal organs (lungs, heart etc).*
- *A cut is made in the chest and stomach and each organ is then removed and examined. Depending on the circumstances a cut may also be made around the head so that the brain can be removed and examined.*
- *In most cases the doctor will remove a small sample of tissue from these organs to examine later under a microscope. The organs are then put back and the cut is closed securely.*
- *Using this approach, in around 30-40% of cases, additional information is found which may help answer the questions that parents and doctors may have.*
- *This type of autopsy is considered the ‘standard’ as it is the usual procedure and may provide most information.*

1. Did or would you agree to a complete autopsy examination following the loss of your baby or child?

Yes ☐ No ☐ Not sure ☐

2. In your opinion, is a complete autopsy examination an acceptable procedure to try and establish cause of death? *(please tick)*

Totally acceptable	Acceptable	No strong opinion	Unacceptable	Totally unacceptable

If you would like to, you can give a fuller explanation of your answer here:

Description of non-invasive autopsy with MRI

- *Recently, a new type of investigation after death has been developed called non-invasive autopsy.*
- *It does not require any cuts to the body. Instead the body is examined using a special type of imaging (like an X ray) called an MRI. There may also be an examination of the placenta or analysis of the baby or child's blood.*
- *Non-invasive autopsy with MRI can provide highly detailed structural information without the need for any 'invasive' sampling and may allow doctors to detect things that are not usually seen at standard autopsy, such as brain abnormalities.*
- *However, in some cases it is not as good as a standard autopsy, for example in the case of an infection, mainly because no tissue is taken to examine under a microscope.*
- *Overall in about 90% of cases a non-invasive autopsy with MRI is as informative as a standard autopsy, but in about 10% of cases a diagnosis may be missed because no tissue has been examined. We also know that non-invasive autopsy is less informative for newborns, infants and children (in about 25% of cases a diagnosis is missed).So there may be additional advantages as well as disadvantages.*

3. If this method was available, would you agree to non-invasive autopsy?

Yes ☐ No ☐ Not sure ☐

4. In your opinion, is a non-invasive autopsy an acceptable procedure to try and establish cause of death? *(please tick)*

Totally acceptable	Acceptable	No strong opinion	Unacceptable	Totally unacceptable

If you would like to, you can give a fuller explanation of your answer here:

Description of minimally invasive autopsy with MRI and tissue sampling

- *Another option being developed is known as minimally invasive autopsy, which includes an MRI, but also includes examination of internal organs and tissue sampling all done using a ‘keyhole surgery’ technique.*
- *A small cut (around 1-2cm) is made to the upper stomach area and a very small camera (like a thin telescope) is inserted into the body which enables the doctor to see the internal organs on a TV monitor.*
- *Using a fine instrument the doctor is also able to remove small pieces of tissue to examine under a microscope if required. The small cut is then closed securely.*
- *This type of autopsy is very new and research is still underway to see how reliable it is. However, because organs can be examined and small pieces of tissue can be taken, it is likely to be almost as reliable as a standard autopsy examination in most cases (we think around 90%-100% as reliable). An MRI may also allow doctors to detect things that are not usually seen at complete autopsy.*
- *There may still be some cases in which a standard autopsy provides most information.*

5. If this method was available, would you agree to minimally invasive autopsy with MRI and tissue sampling?

Yes ☐ No ☐ Not sure ☐

6. In your opinion, is a minimally invasive autopsy an acceptable procedure to try and establish cause of death? *(please tick)*

Totally acceptable	Acceptable	No strong opinion	Unacceptable	Totally unacceptable

If you would like to, you can give a fuller explanation of your answer here:

7. If you could choose between the three methods, which would be your preference?

Complete autopsy examination	☐
Minimally invasive autopsy with MRI and tissue sampling	☐
Non-invasive autopsy with MRI	☐
I wouldn’t choose any of these options	☐
I don’t have a strong preference	☐

Please use the space below to explain your preference.

8. **We know that there are particular aspects about autopsy that are important for parents when they make their decision about whether or not to have one, and which type of autopsy to have. We would like to know how *you* feel about these things. On a scale of 1 to 5 please indicate how important each of the following factors was in your decision.**

	Not at all important				Extremely important
To understand why it happened	1	2	3	4	5
To understand if it might happen again	1	2	3	4	5
To improve medical knowledge	1	2	3	4	5
To prevent this from happening to others	1	2	3	4	5
To help with the grieving process	1	2	3	4	5
To reassure me it was not my fault	1	2	3	4	5
Feeling that my baby/child had ‘suffered enough’	1	2	3	4	5
Not wanting my baby/child to be cut	1	2	3	4	5
Concern about what would happen to the tissue/organs afterwards	1	2	3	4	5
Concern about the baby/child’s appearance afterwards	1	2	3	4	5
Feeling that it would add to my grief	1	2	3	4	5
My religion’s views about autopsy	1	2	3	4	5
Concern it would delay funeral arrangements	1	2	3	4	5
Feeling that I already knew what caused the loss of my baby/child	1	2	3	4	5
Concern about the length of time it may take to get the results	1	2	3	4	5
The complexity and length of the consent form	1	2	3	4	5
The description of autopsy given by the health professional	1	2	3	4	5
Concern that my baby/child might be moved to another hospital	1	2	3	4	5
Other …………………………………………………………	1	2	3	4	5

If you have any comments about any of the issues discussed in this questionnaire, please write them below:

Please complete the following questions to tell us about yourself

9. Age:……………………

10. Sex

Male	
Female	

11. Country you were born in:

……………………………………………

12. Your highest level of education?

No formal qualification	
GCSE or equivalent	
A level or equivalent	
Degree or equivalent	
Postgraduate qualification	

13. What is your ethnicity?

White or White British	
Black or Black British	
Asian or Asian British	
Mixed	
Other:…………………………………	

14. Do you have a religious faith?

Yes	
No	

15. If YES, which faith?

Christian		Sikh	
Muslim		Jewish	
Hindu		Buddhist	
Other		…………………………	

16. Have you ever experienced any of the following? (tick all that apply)

Miscarriage (loss up to 12 weeks gestation)	
Late miscarriage/fetal loss (from 12 to 24 weeks gestation of pregnancy)	
Stillbirth	
Termination for fetal anomaly	
Neonatal/infant death (0-12 months)	
Child death (1-16 years)	
None of the above	

17. If YES, were you approached about an autopsy examination in any of those cases?

Yes	
No	
Not sure	
The coroner’s office issued a compulsory autopsy	

18. If YES, which type of autopsy were you offered (tick all that apply)

Complete autopsy examination	
Limited autopsy – where only certain organs that you consented to are examined	
Minimally invasive autopsy with tissue sampling and MRI	
Non-invasive (external) autopsy with MRI	
Non-invasive (external) autopsy with X-ray	
Not sure	

19. Which type of autopsy did you accept?

Complete autopsy examination	
Limited autopsy	
Minimally invasive autopsy with tissue sampling and MRI	
Non-invasive (external) autopsy with MRI	
Non-invasive (external) autopsy with X-ray	
Not sure	
None – I declined an autopsy	

20. It is difficult to know whether we should ask recently bereaved parents to take part in research because of worry that it will cause extra distress on top of everything you are going through. What has been your experience of taking part in this research?

I'm glad that I took part	
I regret that I took part	
Not sure	

If you would like to add any further comments about your experience of taking part, please write them here:

Thank you for taking the time to complete this questionnaire. Please return it to the health professional or post it back in the freepost envelope enclosed.

✂………

We are also conducting an interview study to help us understand more about people's views of new methods of autopsy. The interview will be between 30-45 minutes, can take place at a location of your choosing such as your home or an office at your hospital. Alternatively it could be done over the telephone. You will receive a £10 gift voucher for taking part. If you are willing to take part in an interview, please leave your contact details and a researcher may contact you (please note not everyone will be contacted):

Name:……………………………………………………………Tel:…………………………………………………………………

Email:……..
