# Supplementary material for: “The communication and support from the health professional is incredibly important”: A qualitative study exploring the processes and practices that support parental decision‐making about postmortem examination
Source: Prenat Diagn. 2019 Nov 4;39(13):1242–53. doi: 10.1002/pd.5575 (PMC6973141; doi:10.1002/pd.5575)
Supplement: Supplementary file 2 — Data S2. Supporting Information [file PD-39-1242-s002.pdf]

**Study design**

This qualitative study conducted in the UK comprises data gathered from 1) a cross-sectional survey about PM with bereaved parents that included space for free-text comments, 2) interviews with a subset of survey respondents, 3) interviews with health professionals, and 4) a focus group with parent advocates. The aim of the study was to examine stakeholders’ views, experiences and acceptability of standard PM as well as less invasive methods of PM. In this paper, however, we have focused on standard PM.

#### **1. Cross-sectional survey with bereaved parents**

A survey (supplementary material) exploring parental views towards PM was developed specifically for this study and co-designed with an advisory team comprising a clinical psychologist, pathologist, radiologist, fetal medicine consultant, genetic counsellor, social scientist, parent advocates and four bereaved parents. The survey presented a brief description of standard PM (systematic examination of all internal organs) as well as less invasive methods (minimally invasive PM using a laparoscopic approach and non-invasive PM using imaging only). For each method participants were asked to respond to questions on likely uptake and acceptability. They were also asked to state their preference between the different methods. Following each set of questions, participants were invited to provide free-text comments explaining their response. Responders were also asked to complete a set of demographic questions and questions to assess their loss and PM experience. At the end of the survey participants could either remain anonymous or choose to provide contact details if they wished to take part in a telephone interview. The survey was made available through the online survey website SurveyMonkey (Survey Monkey Inc, Palo Alto, California, USA) as well as in paper format. Recruitment into the survey was conducted both retrospectively and prospectively.

**2. Qualitative interviews with bereaved parents**

A topic guide was co-designed with input from the advisory team, the aim of which was to explore the findings from the quantitative survey in more depth. The topic guide explored parents’ experience of being approached about standard PM including what support they received when making a decision (for those for whom a Coroner’s PM was not required), reasons for accepting or declining PM, as well as their views on the acceptability of less invasive approaches.

**3. Qualitative interviews with health professionals**

The semi-structured topic guide explored health professionals’ experience of discussing PM with parents and the perceived barriers and facilitators. They also explored views towards less invasive methods and implementation into clinical practice.

#### **4. Focus group with parent advocates**

The semi-structured topic guide explored perceived barriers and facilitators to parental consent for PM and what was considered ‘good practice’ in terms of support and information. They also explored the perceived impact and acceptability of less invasive PM.

**Recruitment**

#### **1. Cross-sectional survey with bereaved parents**

###### **Retrospective recruitment**

Bereaved parents were recruited retrospectively through the following support groups: Antenatal Results and Choices, Sands, The Lullaby Trust and Child Bereavement UK, between June and September 2016. Antenatal Results and Choices supports parents throughout antenatal testing and its consequences including termination of pregnancy for a fetal anomaly; Sands supports parents who have experienced loss at any gestation or after birth, including stillbirth and neonatal death and raises awareness of perinatal mortality; The Lullaby Trust supports bereaved parents and raises awareness of sudden infant death syndrome, and Child Bereavement UK supports families when a baby or child of any age dies or is dying. Anyone who had experienced the loss of a pregnancy (either through miscarriage, termination of pregnancy for a fetal abnormality) or had experience of a perinatal or infant death was eligible to take part irrespective of whether they had been offered a PM or a PM had been requested by the coroner’s office.

###### **Prospective recruitment**

Between September 2016 and December 2017 183 bereaved parents were prospectively recruited through the fetal medicine unit, delivery unit or neonatal intensive care unit of seven hospitals across England. Women and their partners, who were 18 years of age and over and had experienced loss of pregnancy (as described above) or perinatal or infant death, were eligible to participate in the study. Participants were recruited into the study by a member of the healthcare team (such as an obstetrician, bereavement midwife or ICU consultant) following the autopsy examination discussion, irrespective of whether they consented or declined. Potential participants were briefly informed about this study, and if they were interested in taking part or finding out more, given a study pack containing a participant information sheet, survey and freepost envelope. The participant information sheet also included an online link to the survey.

**2. Qualitative interviews with bereaved parents**

A sub-set of survey responders who had indicated their willingness to take part in a telephone interview, were purposively sampled to ensure a range in terms of their experience, whether they consented to a PM procedure or not, and demographics. No time-limit was set in terms of how many months prior to the interview the loss occurred. Interviews were conducted by MR (ClinPsyD, female clinical psychologist, 9 years’ experience in research) or CL (PhD, female senior social scientist, 10 years; experience in research) - trained qualitative researchers with experience conducting interviews on sensitive topics - between November 2016 and May 2017. Neither of the interviewers knew the interview participants.

**3. Qualitative interviews with health professionals**

Health professionals across the UK from a range of clinical backgrounds whose roles include being involved in discussions with parents about PM examination or conducting or interpreting PM results, were identified by the authors, purposively sampled and invited via email to participate in the study. The interviews were conducted by CL either face-to-face or by telephone between April 2016 and July 2017.

#### **4. Focus group with parent advocates**

A focus group was conducted with parent advocates from the four support groups involved in retrospective recruitment for the survey in September 2016. The focus group was facilitated by CL. For all interviews and focus groups written consent was sought to digitally record the discussions, transcribe them verbatim and use anonymised quotes in papers or reports.
